# Ultrasonography characteristics of cystic components in primary salivary gland tumors

**DOI:** 10.1186/s12885-023-11331-1

**Published:** 2023-09-06

**Authors:** AngAng Ding, Huan Lv, Jinye Cao, Xin Wang, Ping Xiong

**Affiliations:** 1 Department of Ultrasound, Shanghai Ninth People’s Hospital, Shanghai Jiao Tong University School of Medicine, Shanghai, 200011 China; 2 Department of Vascular Surgery, Shanghai Ninth People’s Hospital, Shanghai Jiao Tong University School of Medicine, Shanghai, 200011 China

**Keywords:** Salivary gland tumor, Cystic component, Ultrasonography, Pleomorphic adenoma, Warthin tumor

## Abstract

**Objectives:**

The present study aimed to characterize the ultrasonography (US) features of cystic components in salivary gland tumors (SGTs).

**Materials and methods:**

A total of 207 patients (218 lesions) with pathologically confirmed primary SGTs were analyzed. Preoperative US revealed the presence of cystic components in lesions. Lesion size, shape, margin, and US findings of the cystic components, including number, distribution, margin, occupying rate, and internal characteristics, were evaluated.

**Results:**

Similarities were observed between the US performance of benign SGTs (B-SGTs) and malignant SGTs (M-SGTs) with cystic components. Differences in sex and age of patients, number, distribution, and internal characteristics of cystic components were statistically significant. For SGTs with cystic components, the proportions of M-SGTs to ill-defined margins (P = 0.002), eccentric distribution (P = 0.019), and none of the internal characteristics (P = 0.019) were significantly higher than those of B-SGTs. Younger age (P = 0.001), eccentric distribution (P = 0.034) and ill-defined margin (P < 0.001) were risk factors for diagnosing M-SGTs. Cystic component features needed to be combined with lesion indicators (border and shape) to improve diagnostic sensitivity.

**Conclusions:**

US features of the B-SGTs and M-SGTs were significantly different. Cystic component is of interest in the US-related differential diagnosis of B-SGT and M-SGT.

**Clinical relevance:**

Cystic components are potentially valuable in the differential diagnosis of B-SGTs and M-SGTs on US.

**Supplementary Information:**

The online version contains supplementary material available at 10.1186/s12885-023-11331-1.

## Introduction

Salivary gland tumors (SGTs) are rare, representing 2-6.5% of all head and neck neoplasms [1]. The parotid gland is the most common site, followed by the submandibular gland. Benign SGTs (B-SGTs) are predominantly represented by pleomorphic adenoma (PA) and Warthin’s tumor (WT). Malignant SGTs (M-SGTs) are mainly represented by mucoepidermoid carcinomas and adenoid-cystic carcinoma [2]. Accurate predictions of the histopathologic characteristics of tumors along with radiologic findings are useful for adequate surgical planning, especially for avoiding unnecessary surgeries and consequent complications [3].

Ultrasonography (US) is readily available, cost-effective, and widely accepted by patients. US is considered a basic examination for the preoperative assessment of SGTs. US findings for the differentiation of B-SGTs and M-SGTs have been well described, primarily emphasizing the shape, margins, lymph node enlargement, and vascularity [4]. However, the sensitivity of US is low [5].

Cystic components have been reported as common imaging features of SGTs [6, 7]. Magnetic resonance imaging (MRI) manifestations of cystic components in benign and malignant tumors differ [8]. No studies have compared the US manifestations of cystic components in SGTs. This study aimed to describe the US features of cystic components in SGTs and determine whether these features can be useful as indicators for differential diagnosis.

## Materials and methods

**Fig. 1:**
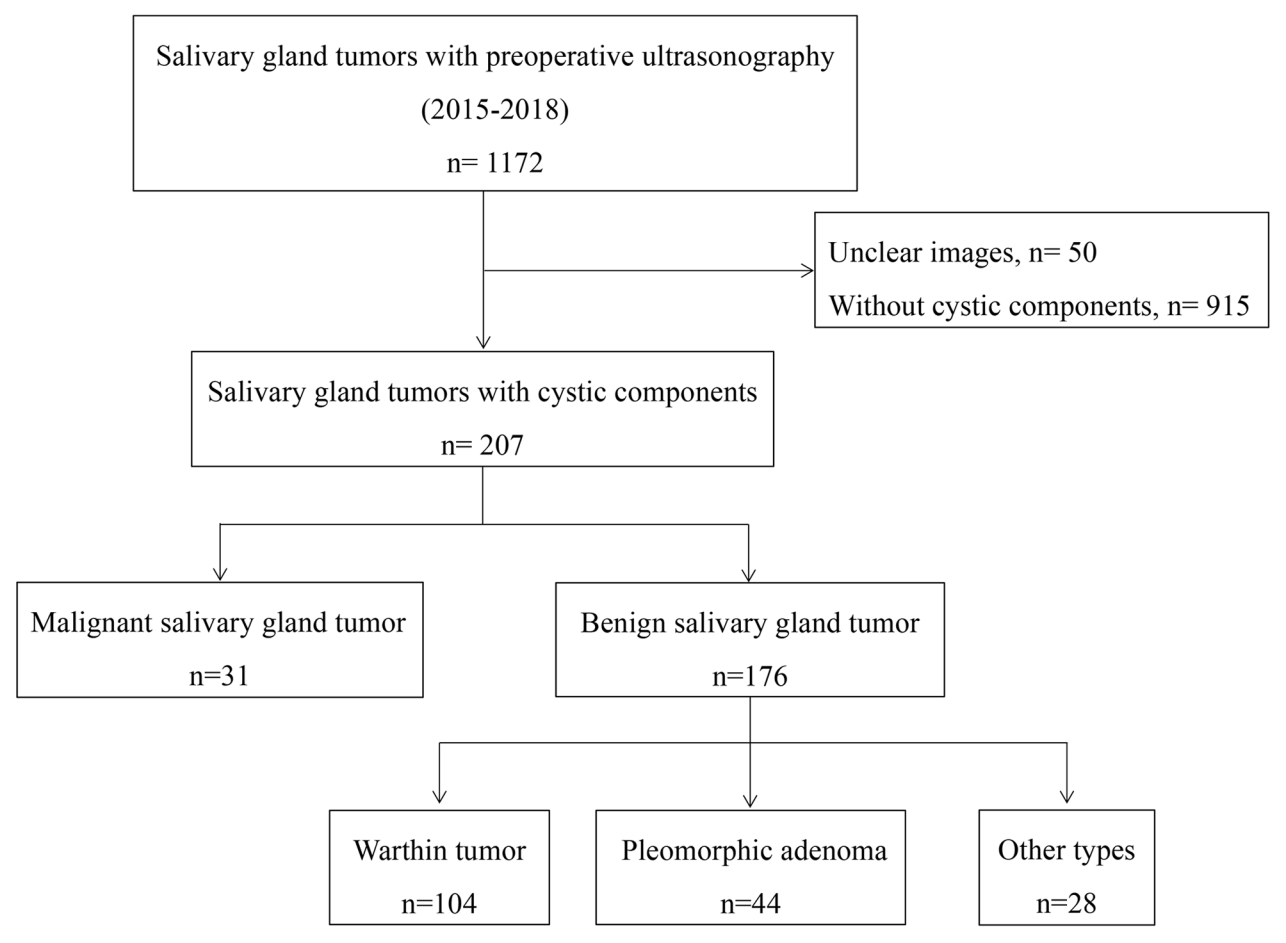
Study algorithm for patient selection

We reviewed the patients’ medical data and identified 1172 patients (1210 lesions) with histopathologically confirmed primary SGTs who had received preoperative US between January 2015 and 2018. The US images stored in the picture archiving and communication system were reviewed, and 207 patients (218 lesions) with cystic components were included in this study (Fig. 1). Patients without cystic components and those with unclear US findings were excluded.

### Imaging methods

US was performed using an Aplio 500 (Toshiba, Tokyo, Japan) and MyLab Twice (Esaote, Italy) systems. The transducers used were 14L5 and LA533. Data from the first preoperative examination were considered. Two certified radiologists with experience in head and neck imaging independently reviewed the US images. They were blinded to the pathological types. Disagreements regarding the imaging features were resolved by consensus.

### Preoperative evaluations

Clinical characteristics included sex, age, region, pathological type. Tumor size was considered to be the maximal length of the transverse section. Lesion shapes were divided into regular (round and oval) and irregular (including lobulated). The margin characteristics were classified as well-defined or ill-defined from the surrounding normal gland tissue.

Cystic components were defined as echoless on gray-scale US. Evaluated imaging findings of cystic components included number (single or multiple), distribution, margin, occupying rate, and internal characteristics.

**Fig. 2:**
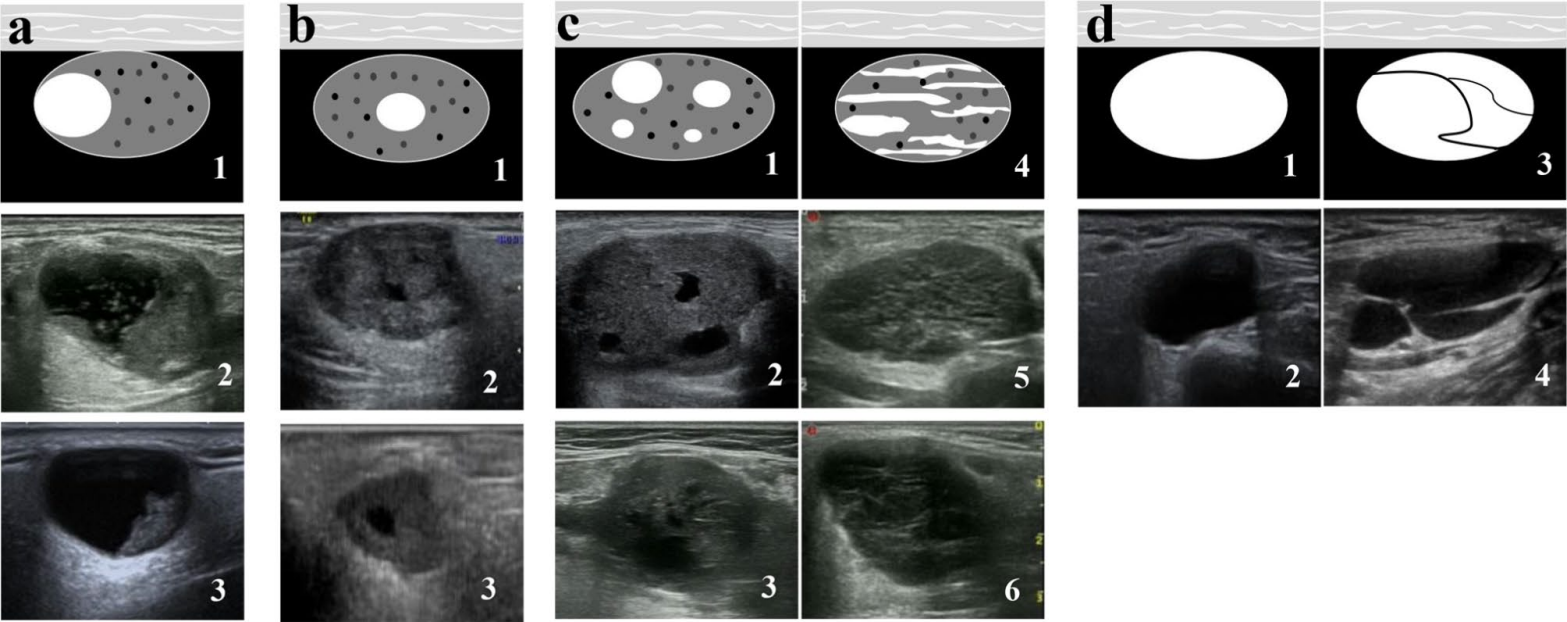
Distribution patterns of cystic components in salivary gland tumors. Four patterns were observed: eccentric (a1-3), central (b1-3), scattered (c1-6), and entire (d1-4). For scattered pattern, c1-3 represent tumors with a few small circular anechoic areas, c 4–6 represent tumors with a sponge-like appearance. For entire pattern, d3-4 represent lesion with hyperechoic separation. a2, b2 and c2, pleomorphic adenoma; a3, mammary analog secretory carcinoma; b3, acinar cell carcinoma. c3, adenoid-cystic carcinoma; c5 Warthin tumor; c6, lymphoepithelial carcinoma. d2 and d3, cystadenoma

Distribution was classified by location: eccentric, central, scattered, and entire (Fig. 2). The scattered distribution comprised two types: tumors with a few small circular anechoic areas and tumors with a sponge-like appearance, in which large and small anechoic areas are finely mixed within the solid part. The entire distribution comprised two other types: entire tumor cystic and hyperechoic separation evident inside.

The occupation rate was categorized into four grades (1–4) as follows:1, small occupying area of 1–25% of the tumor; 2, moderate (26–50%); 3, extensive (51–75%); and 4, diffuse (76–100%).

### Statistical analyses

Statistical analyses were performed using SPSS Statistics for Windows version 28.0 (IBM Corp., Armonk, NY, USA). Student’s t-test, Mann-Whitney U test, Pearson’s χ^2^ test, and Fisher’s exact test were used to analyze the US findings of cystic components in SGTs. The Hosmer-Lemeshow test was used to assess the goodness of fit of the logistic regression model. *P* < 0.05 indicated statistical significance.

## Results

Thirty-one patients with M-SGTs and 176 with B-SGTs were included. Eleven patients with B-SGTs had two masses; in all cases, both masses were Warthin tumors. None of the patients with M-SGT had multiple lesions.

### Clinical characteristics

Eighteen types of SGTs underwent preoperative US. These included seven types of benign tumors and 11 types of malignant tumors (Supplementary Table 1). Oncocytoma, myoepithelioma, poorly differentiated carcinoma, and sebaceous carcinoma displayed no cystic components by US. The proportion of cystic components in M-SGTs (31/135, 22.9%) was slightly higher than that in benign tumors (187/1025, 18.2%), but the difference was not statistically significant (Pearson’s χ^2^ test, *P* = 0.187).

Among B-SGTs, lymphadenomas had the highest frequency (1/1, 100%) of cystic components. Warthin tumors (115/187, 61.5%) accounted for the highest proportion of B-SGTs with cystic components. Among M-SGTs, acinar cell carcinoma had the highest frequency (7/14, 50%) of cystic components. Mucoepidermoid carcinomas (9/31, 29%) accounted for the highest proportion of M-SGTs with cystic components. Relevant clinical findings are described in Table 1.

**Table 1:** Clinical characteristics of salivary gland tumor

Variables	M-SGTs	B-SGTs	*P* value
n	31	176	
Sex^*^			0.024
Male	18	137	
Female	13	39	
Age (y)^*^			< 0.001
Average ± SD	43.7 ± 3.5	56.0 ± 1.0	
Region			0.507
Parotid gland	28	175	
Submandibular gland	3	12	

M-SGT, malignant salivary gland tumor; B-SGT, Benign salivary gland tumor

^*^ difference was statistically significant

The proportion of males with B-SGTs was significantly higher than those with M-SGTs. The difference between males and females was statistically significant (Pearson’s χ^2^ test, *P* = 0.024). The age of onset for M-SGTs was significantly younger than that for B-SGTs (Mann-Whitney U test, *P* < 0.001). The vast majority of primary M-SGT and B-SGT events occurred in the parotid gland (90.3% and 93.6%, respectively). Occurrence in the submandibular glands was rare (Pearson’s χ^2^ test, *P* = 0.507).

### Gray-scale US characteristics

The gray-scale US characteristics of M-SGTs and B-SGTs are summarized in Table 2. Patients with multiple masses were evaluated. The lesion size of M-SGTs was slightly smaller than that of B-SGTs (11–51 vs. 9–73 mm; Student’s t-test, *P* = 0.452). Most M-SGTs and B-SGTs had multiple cystic components (61.3% and 65.2%, respectively). No significant difference was detected between M-SGTs and B-SGTs in the number of cystic components (Fisher’s exact test, *P* = 0.670).

**Table 2:** Imaging findings of cystic components

Variables	M-SGT	B-SGT	*P* value
n	31(100)	187 (100)	
Lesion Size (mm)	28.0 ± 1.9	29.6 ± 0.8	0.452
Number			0.670
Single	12 (38.7)	65 (34.8)	
Multiple	19 (61.3)	122 (65.2)	
Margin^*^			0.002
Well-defined	14 (45.2)	137 (73.2)	
Ill-defined	17 (54.8)	50 (26.7)	
Distribution^*^			0.019
Eccentric	16 (51.6)	64 (34.2)	
Central	7 (22.6)	21 (11.2)	
Scattered	8 (25.8)	91 (48.6)	
Entire	0	11 (5.9)	
Occupying rate			0.786
1	19(61.3)	101 (54.0)	
2	6(19.4)	35 (18.7)	
3	2 (6.5)	22 (11.8)	
4	4(12.9)	29 (15.5)	
Internal characteristic^*^			0.019
Present	12 (38.7)	115 (61.5)	
None	19 (61.2)	72 (38.5)	

M-SGT, malignant salivary tumor; B-SGT, Benign salivary tumor;

^*^ Difference was statistically significant

**Fig. 3:**
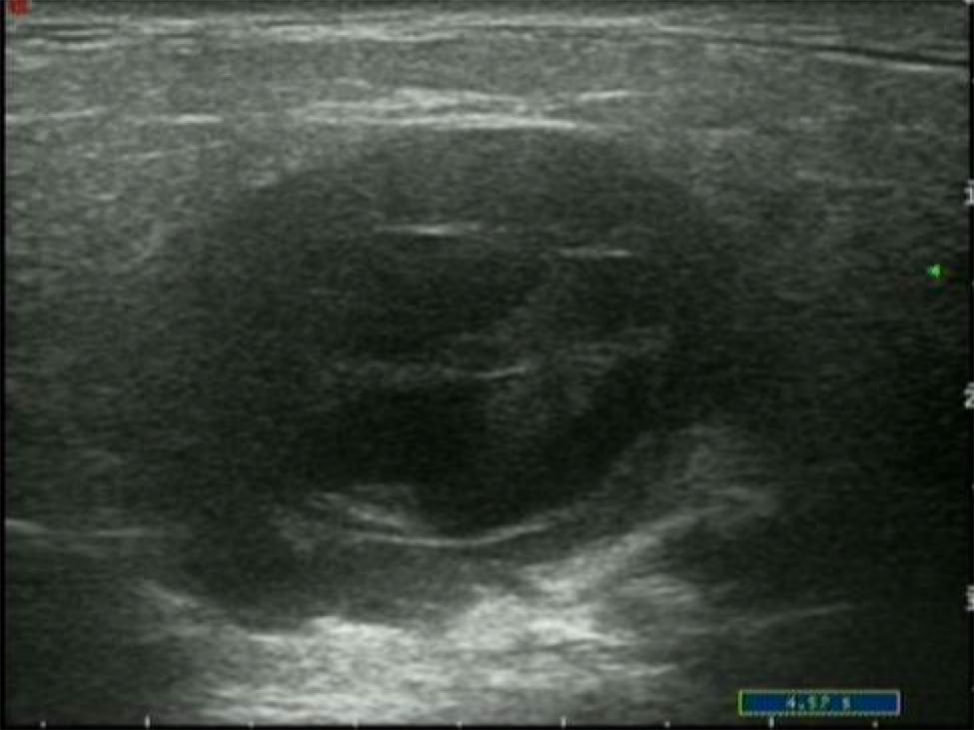
Basal cell adenocarcinoma with ill-defined cystic components on US

A significant difference was noted between the cystic components of M-SGTs and B-SGTs with respect to their distribution (Fisher’s exact test, *P* = 0.019). The proportion of M-SGTs showing an eccentric distribution was significantly higher than that of the B-SGTs (51.6% vs. 34.2%). M-SGTs and B-SGTs differed in their cystic component margin characteristics. Most M-SGTs (54.8%) exhibited an ill-defined border (Fig. 3), whereas most B-SGT lesions (73.2%) had well-defined margins. A significant difference was observed (Pearson’s χ^2^ test, *P* = 0.002). Most M-SGT and B-SGT lesions had a low occupying rate of cystic components (61.3% and 54%, respectively), with no significant difference (Pearson χ^2^ test, *P* = 0.786).

**Fig. 4:**
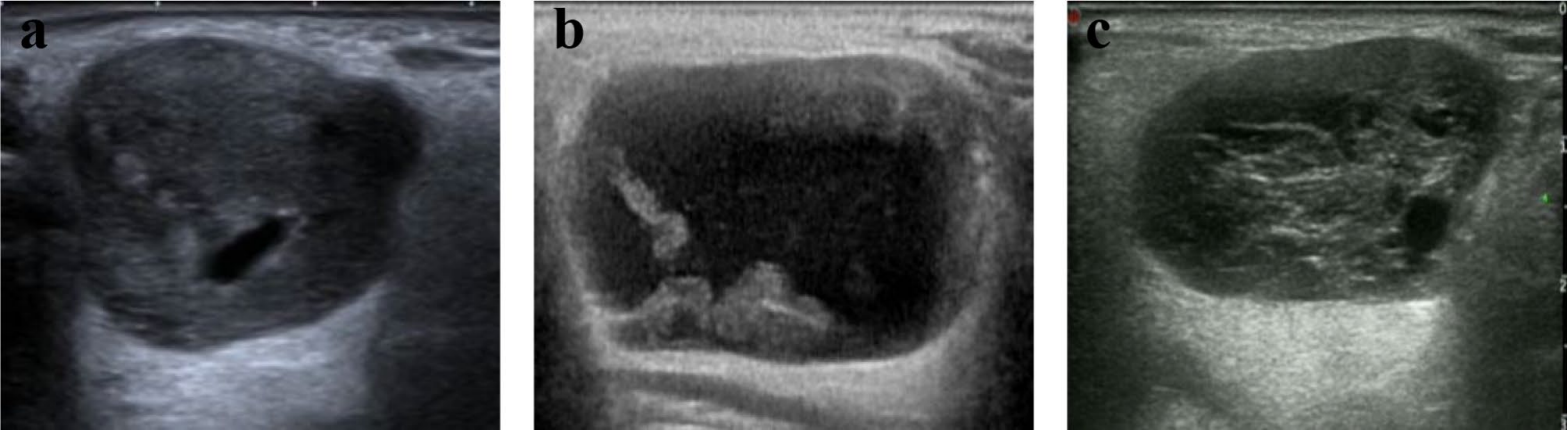
Internal characteristics of cystic components in SGTs. a, Pleomorphic adenoma with no internal characteristic. b, Warthin tumor with papillary structure. c, Warthin tumor with spongiform cyst

The evaluated internal characteristics included papillary structures and spongiform cysts (Fig. 4). Most M-SGT lesions lacked these characteristics (61.2%), while the majority of B-SGT lesions (61.5%) had one of the two features (Supplementary Table 2). A significant difference was evident (Pearson’s χ^2^ test, *P* = 0.019).

### Multi-factor analysis

Based on the above results, sex, age, distribution, margin, and internal characteristics were used as variables for binary logistic regression analysis. The outcome was benign or malignant. Three indicators were statistically significant: age (*P* = 0.001), eccentric distribution (*P* = 0.034), and ill-defined margin (*P* < 0.001, Table 3). Younger age, eccentric distribution and ill-defined margin were risk factors for diagnosing M-SGTs. The Hosmer-Leme show goodness of χ^2^ fit value was 12.889 (*P* = 0.116).

**Table 3:** Odds ratio and 95% confidence interval of univariant and multivariant analysis

Variables	Univariant		Multivariant	
	OR (95% CI)	P value	OR (95% CI)	P value
Age	0.953 (0.931–0.977)	< 0.001	0.953 (0.927–0.980)	0.001
Sex	0.388 (0.175–0.862)	0.02	0.667 (0.259–1.723)	0.403
Distribution		0.073		0.192
Eccentric	2.844 (1.148—7.043)	0.024	4.095 (1.113–15.065)	0.034
Central	3.792 (1.237–11.619)	0.02	4.226 (0.875–20.407)	0.073
Entire	0.000	0.999	0.000	0.999
Margin	3.327 (1.528–7.243)	0.02	10.03 (3.377–29.787)	< 0.001
Internal characteristics	0.395 (0.181–0.863)	0.02	0.942 (0.332–2.673)	0.911

OR, Odds ratio; CI, Confidence interval

### Efficacy of US features in diagnosis of M-SGTs

In terms of the diagnostic efficacy of cystic components, the sensitivity and specificity for diagnosing M-SGTs alone were low (Table 4). In addition, the ability of the lesion features of shape and margin in combination with cystic components to diagnose M-SGTs was assessed. When two or more of the indicators were present, the sensitivity of the diagnosis was highest (83.9%). An ill-defined margin of the lesion had the highest specificity (98.9%) for diagnosing malignancy.

**Table 4:** Efficacy of image features in the diagnosis of M-SGTs

Feature	Sensitivity	Specificity
Cystic components		
Ill-defined margin	54.8%	73.2%
Eccentric distribution	51.6%	65.8%
No internal characteristic	61.3%	61.5%
Lesion		
Ill-defined margin	64.5%	98.9%
Irregular shape	35.5%	98.4%
Two or more above features	83.9%	73.8%

M-SGT, malignant salivary tumor

### Cystic components in PAs and WTs

The cystic components in PAs and WTs were significantly different in the US (Table 5). Compared with PA, most WT cystic components had multiple (80.9% and 31.8%, respectively), scattered distribution (73.9% and 11.4%, respectively), and internal characteristics (87.8% and 9.1%, respectively). The probability of an ill-defined margin (38.3% and 6.8%, respectively) was greater than that of PA. Moreover, the proportion of small occupying rate of cystic components (< 25%) in WTs was smaller than that in PAs (52.3%, and 75%, respectively).

**Table 5:** Cystic components in pleomorphic adenoma and Warthin tumor

Variables	PA	WT	P value
n	44	115	
Number^*^			< 0.001
Single	30 (68.2)	22 (19.1)	
Multiple	14 (31.8)	93 (80.9)	
Margin^*^			< 0.001
Well-defined	41(93.2)	71 (61.7)	
Ill-defined	3 (6.8)	44 (38.3)	
Distribution^*^			< 0.001
Eccentric	29 (65.9)	24 (20.9)	
Central	10 (22.7)	5 (4.3)	
Scattered	5 (11.4)	85 (73.9)	
Entire	0	1 (0.9)	
Occupying rate^*^			0.021
1	33 (75)	60 (52.3)	
2	5 (11.4)	26 (22.6)	
3	1 (2.3)	18 (15.7)	
4	5 (11.4)	11 (9.6)	
Internal characteristic^*^			< 0.001
Papillary structure	4 (9.1)	23 (20)	
Spongiform cyst	0	78 (67.8)	
None of the above characteristics	40 (90.9)	14 (12.2)	

PA, Pleomorphic adenoma; WT, Warthin tumor;

^*^ Difference was statistically significant

## Discussion

US can provide excellent tissue characterization, multiplanar information, and vascular pattern for superficial tumors within the parotid or submandibular glands [9, 10]. Reports on the US performance of SGTs have mainly focused on the shape, margin, echogenicity, echotexture, posterior echo, and blood flow. However, the sensitivity is low [5], possibly because low-grade M-SGTs may exhibit US characteristics of benign tumors [11, 12]. Herein, we aimed to identify new features to improve the diagnostic efficiency of US.

Cystic components have been reported as common imaging features of SGTs, such as WTs [6], basal cell adenomas [7], cystadenoma [13], mucoepidermoid carcinomas [14], acinic cell carcinomas, and mammary analog secretory carcinoma [15]. The proportion of cystic components in B-SGTs can reach 79% [8]. US is a quick method to differentiate solid from cystic lesions in the superficial areas of the head and neck [10]. The 4th World Health Organization classification issued in 2017 defines 11 different types of benign epithelial salivary tumors and 22 carcinomas. In the present study, we retrospectively found that 18 types of epithelial SGTs underwent preoperative US between January 2015 and January 2018. Of these, 14 types of SGTs had cystic components (five B-SGTs and nine M-SGTs). Among them, WT was the most common type.

Given the prevalence of cystic components in SGTs, we summarized the US performance of cystic components of B-SGTs and M-SGTs and clarified whether they could provide effective information for the differential diagnosis of the two. We found that the proportion of cystic components differed in SGTs of different pathological types. The US features of the B-SGTs and M-SGTs were significantly different. The cystic components in M-SGTs had a higher probability of displaying ill-defined, eccentric, and no internal features than B-SGTs. Despite the low sensitivity of cystic components as the sole means of diagnosing M-SGT, these components remain potential indicators for differential diagnosis. When combined with US features of the lesion, including border and shape, the diagnostic efficiency can be significantly improved.

Most studies on the imaging findings of SGTs only determined the presence or absence of cystic components within the lesions. Studies of the imaging features of cystic components are rare. It has been reported that cystic components of PAs and WTs can be classified into various patterns on US [16]. More importantly, imaging manifestations of cystic components in benign and malignant tumors differ. Irregular margins of cystic components are more frequently observed in M-SGTs than in B-SGTs [8]. This is consistent with the present observation of the significantly higher proportion of ill-defined borders in M-SGTs than in B-SGTs, and the association of the presence of these borders as a risk factor for M-SGTs. However, in terms of distribution, M-SGTs appeared to have the highest proportion of eccentric distribution, which is also a risk factor for diagnosing M-SGTs. The central distribution was not unique to M-SGTs, in contrast to an earlier study [8]. This may reflect the different grouping criteria used in the two studies. The present study divided the distribution into four types, while the distribution of the prior study comprised three groups.

Pathologically, multicystic and papillary growth patterns, cystic changes, necrosis, and hemorrhage in solid SGTs are responsible for the cystic components of tumors [17]. The imaging differences are thought to result from histological differences. We evaluated the internal characteristics and found a correlation between US and pathological findings. On US, the majority of WTs displayed papillary bulges or spongiform cysts (87.8%), which may be related to the slit-like space and papillary structures protruding into the cystic cavity of WTs on histopathology. For cystadenomas, most lesions displayed features of papillary bulges, which should also correlate with the pathological features of intraluminal papillary proliferation. Notably, marked differences were observed in the internal characteristics of benign and malignant tumors. Most M-SGT lesions showed no internal features. This may be because the cystic components of malignant tumors usually result from necrosis or hemorrhage [8, 18].

Most (80–85%) parotid gland tumors are benign, and most are PAs and WTs [19]. These two tumors have different malignant transformation probabilities and treatment methods. Preoperative differential diagnosis is important [20]. Cystic areas within lesions were reportedly detected in 20.8% of PAs and 45.2% of WTs [21]. Therefore, because of the predominant incidence of PA and WT in SGTs, we performed a separate analysis of the cystic components and found significant differences. The differences were statistically significant across all assessed features. Thus, cystic components can be definitive indicators for the differential diagnosis between the two. The occurrence of M-SGTs is relatively infrequent, encompassing a spectrum of eleven distinct pathological subtypes exhibiting cystic components in our study. It is noteworthy that the sample size for each specific M-SGT subtype was below ten cases, consequently precluding a comparative investigation.We further compared the effectiveness of cystic component features in the diagnosis of M-SGTs, including ill-defined margins, eccentric distribution, and absence of internal characteristics. However, their sensitivity and specificity for diagnosing M-SGTs alone are not ideal. Given the important role of lesion margins and shapes in the differentiation of benign and malignant tumors [4], we further evaluated the diagnostic efficacy of combining lesion features with cystic component features. The sensitivity significantly increased. Thus, cystic components are potentially valuable in the differential diagnosis of B-SGTs and M-SGTs on US. Comprehensive evaluations of lesions are needed for a definitive conclusion.

The present study clarified the US performance of cystic components in SGTs. The study has two limitations. First, it was a single-center, retrospective study. US features were analyzed based on storage images and were not evaluated in real-time. In real-time US, cystic components may appear as fluttering scattered hyperechoic spots, whereas on still images, they may appear as isoechoic areas that cannot be distinguished from solid components on retrospective analysis. Therefore, our study may have underestimated the proportion of SGTs with cystic degeneration. Second, there was a lack of comparison with other imaging methods such as MRI.

## Conclusion

This study is the first-known comparison of the US performance of cystic components in SGTs. The study included the evaluation of multiple characteristics. Margin, distribution, and internal characteristics were the key characteristics. On US, cystic components are of interest in the differential diagnosis of B-SGT and M-SGT. A comprehensive evaluation of the lesion is needed to improve the sensitivity of the diagnosis.

### Electronic supplementary material

Below is the link to the electronic supplementary material.

Supplementary Material 1

Supplementary Material 2

## Data Availability

All data generated or analysed during this study are included in this published article and its supplementary information files.
