# Supplementary material for: Ultrasonography characteristics of cystic components in primary salivary gland tumors
Source: BMC Cancer. 2023 Sep 6;23:833. doi: 10.1186/s12885-023-11331-1 (PMC10481467; doi:10.1186/s12885-023-11331-1)
Supplement: Supplementary file 1 — Supplementary Material 1 [file 12885_2023_11331_MOESM1_ESM.docx]

Supplementary Table 1. Summary of cystic lesions in the salivary gland tumor

| Pathological type | Total | Presence of cystic components | Proportion (%) |
| --- | --- | --- | --- |
| Benign | 1025 | 187 (18.2) | 100 |
| Pleomorphic adenoma | 624 | 44 (7.1) | 23.5 |
| Warthin tumor | 314 | 115 (33.7) | 61.5 |
| Basal cell adenoma | 60 | 13 (21.7) | 7.0 |
| Cystadenoma | 19 | 14 (73.9) | 7.5 |
| Lymphadenoma | 1 | 1 (100) | 0.5 |
| Oncocytoma | 4 | 0 | / |
| Myoepithelioma | 3 | 0 | / |
| Malignant | 135 | 31(22.9) | 100 |
| Mucoepidermoid carcinoma | 34 | 9 (26.5) | 29.0 |
| Carcinoma in pleomorphic adenoma | 22 | 2 (9.1) | 6.5 |
| Adenoid cystic carcinoma | 18 | 2 (11.1) | 6.5 |
| Lymphoepithelial carcinoma | 16 | 4 (25) | 12.9 |
| Acinar cell carcinoma | 14 | 7 (50) | 22.6 |
| Squamous cell carcinoma | 8 | 1 (12.5) | 3.2 |
| Adenocarcinoma，NOS | 8 | 1 (12.5) | 3.2 |
| Mammary analogue secretory carcinoma | 7 | 3 (42.9) | 9.7 |
| Basal cell adenocarcinoma | 5 | 2 (40) | 6.5 |
| Poorly differentiated carcinoma | 2 | 0/2 | / |
| Sebaceous carcinoma | 1 | 0/1 | / |
