# Supplementary material for: Ultrasonography characteristics of cystic components in primary salivary gland tumors
Source: BMC Cancer. 2023 Sep 6;23:833. doi: 10.1186/s12885-023-11331-1 (PMC10481467; doi:10.1186/s12885-023-11331-1)
Supplement: Supplementary file 2 — Supplementary Material 2 [file 12885_2023_11331_MOESM2_ESM.docx]

**Supplementary Table 2. Internal characteristics of SGTs of each pathological type**

| Pathological type | Papillary structure | spongiform cyst | None of the above characteristics |
| --- | --- | --- | --- |
| Pleomorphic adenoma | 4 | 0 | 40 |
| Warthin tumor | 23 | 78 | 14 |
| Basal cell adenoma | 1 | 0 | 12 |
| Cystadenoma | 8 | 0 | 6 |
| Lymphadenoma | 1 | 0 | 0 |
| Mucoepidermoid carcinoma | 3 | 2 | 4 |
| Carcinoma in pleomorphic adenoma | 1 | 0 | 1 |
| Adenoid cystic carcinoma | 0 | 1 | 1 |
| Lymphoepithelial carcinoma | 0 | 2 | 2 |
| Acinar cell carcinoma | 1 | 0 | 6 |
| Squamous cell carcinoma | 0 | 0 | 1 |
| Adenocarcinoma，NOS | 0 | 0 | 1 |
| Mammary analogue secretory carcinoma | 2 | 0 | 1 |
| Basal cell adenocarcinoma | 0 | 0 | 2 |
